# A randomized trial of iron isomaltoside versus iron sucrose in patients with iron deficiency anemia

**DOI:** 10.1002/ajh.24633

**Published:** 2017-02-01

**Authors:** Richard Derman, Eloy Roman, Manuel R. Modiano, Maureen M. Achebe, Lars L. Thomsen, Michael Auerbach

**Affiliations:** ^1^ Thomas Jefferson University Philadelphia Pennysylvania USA; ^2^ Lakes Research Miami Lakes Florida USA; ^3^ ACRC Arizona Clinical Research Tucson Arizona USA; ^4^ Division of Hematology Brigham and Women's Hospital, Dana Farber Cancer Institute Boston Massachusetts USA; ^5^ Department of Clinical and Non‐clinical Research Pharmacosmos A/S Holbaek Denmark; ^6^ Georgetown University School of Medicine Washington District of Columbia USA

## Abstract

Iron deficiency anemia (IDA) is common in many chronic diseases, and intravenous (IV) iron offers a rapid and efficient iron correction. This trial compared the efficacy and safety of iron isomaltoside (also known as ferric derisomaltose) and iron sucrose in patients with IDA who were intolerant of, or unresponsive to, oral iron. The trial was an open‐label, comparative, multi‐center trial. Five hundred and eleven patients with IDA from different causes were randomized 2:1 to iron isomaltoside or iron sucrose and followed for 5 weeks. The cumulative dose of iron isomaltoside was based on body weight and hemoglobin (Hb), administered as either a 1000 mg infusion over more than 15 minutes or 500 mg injection over 2 minutes. The cumulative dose of iron sucrose was calculated according to Ganzoni and administered as repeated 200 mg infusions over 30 minutes. The mean cumulative dose of iron isomaltoside was 1640.2 (standard deviation (SD): 357.6) mg and of iron sucrose 1127.9 (SD: 343.3) mg. The primary endpoint was the proportion of patients with a Hb increase ≥2 g/dL from baseline at any time between weeks 1‐5. Both non‐inferiority and superiority were confirmed for the primary endpoint, and a shorter time to Hb increase ≥2 g/dL was observed with iron isomaltoside. For all biochemical efficacy parameters, faster and/or greater improvements were found with iron isomaltoside. Both treatments were well tolerated; 0.6% experienced a serious adverse drug reaction. Iron isomaltoside was more effective than iron sucrose in achieving a rapid improvement in Hb. Furthermore, iron isomaltoside has an advantage over iron sucrose in allowing higher cumulative dosing in fewer administrations. Both treatments were well tolerated in a broad population with IDA.

## 1 Introduction

Iron deficiency anemia (IDA) is a common problem associated with many chronic diseases which include chronic kidney disease (CKD),^1^ cancer,^2^ infections,^3^ chronic heart failure (CHF),^4^ inflammatory bowel disease (IBD),^5^ and bariatric procedures.^6^ It is also common in women who have recently given birth^7^ or suffer from heavy menstrual bleeding.^8^

International guidelines^9^, ^10^, ^11^ recommend IV iron as the preferred option when oral iron was either ineffective or not tolerated, either because of limited absorption, lack of adherence, intolerance, or when the iron need is high. IV iron is considered more effective, better tolerated, and improves quality of life (QoL) to a greater extent than oral iron. Thus, the use of IV iron may result in improved iron correction with better adherence, fewer visits to the medical practitioner, and greater convenience. Iron isomaltoside (also known as ferric derisomaltose) is one of the newer IV iron formulations available. It was initially launched in Europe in 2010 and consists of iron and a carbohydrate moiety where the iron is tightly bound in a matrix structure. It is the matrix structure that enables a controlled and slow release of iron to iron‐binding proteins, avoiding potential toxicity from release of labile iron.^12^ Previous published data demonstrate good safety and efficacy of iron isomaltoside in different populations with different comparators.^13^, ^14^, ^15^, ^16^, ^17^, ^18^, ^19^, ^20^, ^21^, ^22^ A previous trial compared efficacy of ferric carboxymaltose versus iron sucrose in IBD patients,^23^ and iron isomaltoside has been compared with iron sucrose in CKD.^13^ This trial is the first head to head trial of iron isomaltoside against iron sucrose outside the realm of CKD. The objective of the present trial was to compare the efficacy and safety of iron isomaltoside with iron sucrose in patients with IDA over a wide range of different clinical diagnoses.

## 2 Patients and methods

### 2.1 Trial design

This was a prospective, comparative, open‐label, randomized, non‐inferiority multicenter trial incorporating 7–12 visits during a 5‐week period.

The protocol and amendments were approved by the relevant Institutional Review Boards and conducted in accordance with good clinical practice and the Declaration of Helsinki of 1975, as revised in 2008. The trial was registered with ClinicalTrials.gov (NCT02130063). Written informed consent was obtained from all participants.

### 2.2 Participants

The trial was conducted at 48 sites in the United States. Patients ≥18 years of age with moderate‐to‐severe IDA caused by different etiologies, and with a documented history of intolerance of, or unresponsiveness to, oral iron, a Hb <11.0 g/dL, TSAT <20%, and *s*‐ferritin <100 ng/mL were recruited. Inclusion and exclusion criteria are shown in Supporting Information Table SI.

### 2.3 Interventions

Patients were randomized 2:1 to either iron isomaltoside (Monofer^®^, Pharmacosmos A/S, Holbaek, Denmark) or iron sucrose (Venofer^®^, Vifor Pharma, Glattbrugg, Switzerland). The cumulative dose of iron isomaltoside (1000 mg, 1500 mg or 2000 mg) depended on Hb level and body weight; 1000 (Hb ≥10 g/dL for patients weighing <70 kg), 1500 (Hb ≥ 10 g/dL, ≥70 kg or Hb < 10 g/dL, <70 kg), or 2000 (Hb < 10 g/dL, ≥70 kg) mg. Thousand milligrams was administered in a single dose, whereas doses of 1500 and 2000 mg iron were administered in two administrations, one week apart; 1000 + 500 mg or 1000 + 1000 mg respectively. The 1000 mg infusions were diluted in 100 mL 0.9% sodium chloride and given over approximately 15 minutes. The 500 mg bolus injections were administered undiluted over approximately 2 minutes. In line with prescribing information in the labeling of iron sucrose, the cumulative dose of iron sucrose was calculated according to the Ganzoni formula in the following way: Cumulative iron dose (mg)=[body weight (kg)×(target Hb ‐ actual Hb (g/dL)]×2.4+ 500 mg depot iron.^24^ Iron sucrose was administered as an infusion of 200 mg over approximately 30 minutes up to twice weekly, according to the prescribing information.^25^, ^26^ The maximum cumulative dosage of iron sucrose was 2000 mg. During the trial, other iron supplementation than the investigational drug, blood transfusion, and ESAs were proscribed.

### 2.4 Objective and endpoints

The trial was designed with the primary objective to evaluate and compare iron isomaltoside with iron sucrose in its ability to increase Hb in patients with IDA when oral iron formulations were ineffective or could not be used or where there was a clinical need to deliver iron rapidly. The primary efficacy endpoint was the proportion of patients with a Hb increase of ≥2 g/dL from baseline at any time from weeks 1 to 5. The secondary efficacy endpoints included time to Hb increase ≥2 g/dL, and change in Hb, *s‐*ferritin, TSAT, and *s*‐iron, and total quality of life (QoL) score (Short Form 36 (SF‐36) questionnaire). Safety endpoints included the number of patients who experienced any adverse drug reaction (ADR) and safety laboratory assessments (complete hematology, *s‐* sodium, *s‐*potassium, *s‐*calcium, *s‐*phosphate, *s‐*urea, *s‐*creatinine, *s‐*albumin, *s*‐globulin, *s‐* bilirubin, aspartate aminotransferase, alanine aminotransferase, and C‐reactive protein). The primary endpoint was tested for non‐inferiority. Additionally, if the 95% confidence interval (CI) was entirely above 0, this was evidence of statistically significant superiority at the 5% level. The *p*‐value associated with a test of superiority was to be calculated. The remaining endpoints were tested for superiority.

### 2.5 Sample size and randomization

A stratified block randomization methodology was used to assign patients in a 2:1 ratio to receive iron isomaltoside or iron sucrose. The randomization to treatment groups was stratified by screening Hb (Hb < 10.0 g/dL and Hb ≥ 10 g/dL) and origin of disease (oncology, gastroenterology, gynecology, and others).

With a 2:1 randomization and a 2‐sided significance level of 5%, there would be approximately 90% power to demonstrate non‐inferiority when using an absolute non‐inferiority margin of 12.5%‐points. As the trial was designed to demonstrate non‐inferiority, it was a requirement that the analyses of the full analysis set (FAS) and per protocol (PP) population led to similar conclusions. Thus, both analysis sets needed to be powered properly. It was anticipated that approximately 10% would sustain a major protocol deviation, and therefore a total of 500 had to be randomized.

### 2.6 Statistical methods

The following data sets were used in the analyses (Supporting Information Figure S1).

The randomized population (*N* = 511) included those who were randomized in the trial. The safety population (*N* = 501) included randomized patients who received at least one dose of the trial drug. The full analysis set (FAS) population (*N* = 491) included randomized patients who received at least one dose of the trial drug, and had at least one post‐baseline Hb assessment. The PP population (*N* = 454) included all patients in the FAS who did not sustain major protocol deviation of clinical relevance.

The risk difference was used to compare the proportion of patients with an increase in Hb ≥2 g/dL at any time point during the treatment period. Risk difference and the associated 2‐sided 95% Newcombe CI of the difference in percentage of patients were calculated, adjusting for strata using the Cochran‐Mantel‐Haenszel method. Non‐inferiority of iron isomaltoside against iron sucrose could be claimed if the lower bound of the 95% CI was above −12.5%. Superiority could be claimed if the lower bound of the 95% CI was above 0, and a *p*‐value associated with a test of superiority was calculated. The primary analysis was repeated for the PP analysis set. The primary efficacy data were tabulated using number, mean, standard deviation (SD), minimum, maximum, and 95% confidence interval (CI).

A mixed model for repeated measures (MMRM), with treatment, visit, treatment‐by‐visit, and strata as factors and baseline value as covariate, was used to compare the average change in Hb, *s‐*ferritin, TSAT, *s‐*iron, and QoL score. All tests were two‐tailed and the significance level was 0.05. The baseline characteristics and safety data were displayed descriptively.

## 3 Results

### 3.1 Patients

Here 1112 patients were screened of whom 511 were randomized 2:1 to the iron isomaltoside group (342) or iron sucrose group (169). Of the 511 enrolled, 469 (92%) completed the trial. The details of patient disposition are summarized in Supporting Information Figure S1.

The demographics and baseline characteristics are summarized in Table 1. Baseline laboratory variables are shown in Supporting Information Table SII. Overall baseline characteristics were comparable between the treatment groups (Tables 1, Supporting Information Table SII).

**Table 1:** Baseline demographics, full analysis set

	**Iron isomaltoside (*n* = 330)**	**Iron sucrose (*n* = 161)**	**Total (*N* = 491)**
**Age (years)**			
Mean (SD)	49 (16)	47 ( 15)	48 (16)
Median (Min; Max)	45 (19; 95)	44 (19; 87)	45 (19; 95)
**Gender (*N*, %)**			
Women	297 (90.0)	146 (90.7)	443 (90.2)
Men	33 (10.0)	15 (9.3)	48 (9.8)
**Race (*N*, %)**			
White	208 (63.0)	99 (61.5)	307 (62.5)
Black or African American	111 (33.6)	54 (33.5)	165 (33.6)
Asian	2 (0.6)	1 (0.6)	3 (0.6)
Others	9 (2.7)	7 (4.3)	16 (3.3)
**Weight (kg)**			
Mean (SD)	86 (23)	82 (21)	85 (23)
Median (Min; Max)	84 (50; 209)	79 (50; 152)	81 (50; 209)
**Origin of disease causing IDA (*N*, %)**			
Gynecology	158 (47.9)	79 (49.1)	237 (48.3)
Gastroenterology	111 (33.6)	53 (32.9)	164 (33.4)
Oncology	6 (1.8)	3 (1.9)	9 (1.8)
Others	55 (16.7)	26 (16.1)	81 (16.5)

### 3.2 Exposure to iron

A total of 333 patients were dosed with iron isomaltoside and 168 with iron sucrose. The patients received 1 or 2 administrations in the iron isomaltoside group and from 1 to 10 administrations in the iron sucrose group, with the vast majority requiring 5‐9 administrations (> 96%). The mean (SD) planned dose for iron isomaltoside and iron sucrose was 1663.7 (312.1) and 1203.2 (279.7) mg, respectively, and the actual mean (SD) dose was 1640.2 (SD: 357.6) and 1127.9 (343.3) mg, respectively.

### 3.3 Efficacy results

#### 3.3.1 Change in hemoglobin

The primary analysis (proportion with an increase in Hb ≥2 g/dL from baseline at any time from week 1 to week 5) was conducted on the FAS (*N* = 491) and PP analysis set (*N* = 454).

A summary of the primary efficacy analysis in the FAS and PP analysis set is provided in Table 2. There were more responders in the iron isomaltoside group compared with the iron sucrose group, with a risk difference of 16.7%‐points in the FAS and 15.9%‐points in the PP set. Since the lower end of the 95% CI for the risk difference was above −12.5%‐points in both the FAS and PP analysis set, non‐inferiority of iron isomaltoside to iron sucrose could be claimed.

**Table 2:** Analysis of proportion of patients with Hb increase ≥2 g/dL

**Increase in Hb ≥2 g/dL**	**Iron isomaltoside**	**Iron sucrose**
**FAS (*N*, %)**	330 (100.0)	161 (100.0)
Responders, *E/n* (%)	226/330 (68.5)	83/161 (51.6)
Risk difference (95% CI) (%)	16.7 (7.5; 25.7)	
Superiority test, *p*‐value	<0.0001	
**PP analysis set (*N*, %)**	311 (100.0)	143 (100.0)
Responders, *E/n* (%)	218/311 (70.1)	77/143 (53.8)
Risk difference (95% CI) (%)	15.9 (6.3; 25.4)	
Superiority test, *p*‐value	0.0002	

Abbreviations: FAS, full analysis set; PP, per protocol; *N*, number of patients in analysis set; *E*, number of patients who increased in Hb ≥2 g/dL; *n*, number of patients with non‐missing values.

Non‐inferiority could be claimed if the lower bound of the 95% CI was above −0.125.

Risk difference adjusted for strata using the Cochran‐Mantel‐Haenszel method, *p*‐value from a Cochran‐Mantel‐Haenszel Chi‐square test adjusted for strata.

As non‐inferiority was proven, the predetermined test for superiority was performed, which also confirmed superiority of iron isomaltoside compared with iron sucrose (*p* < 0.0001, Table 2).

In the FAS, the largest increase in Hb from baseline to any time from week 1 to week 5 [mean (SD)] was 2.74 (1.32) g/dL in the iron isomaltoside group and 2.20 (1.20) g/dL in the iron sucrose group. Increases in Hb in the PP analysis set were comparable with superiority of iron isomaltoside over iron sucrose.

**Figure 1:**
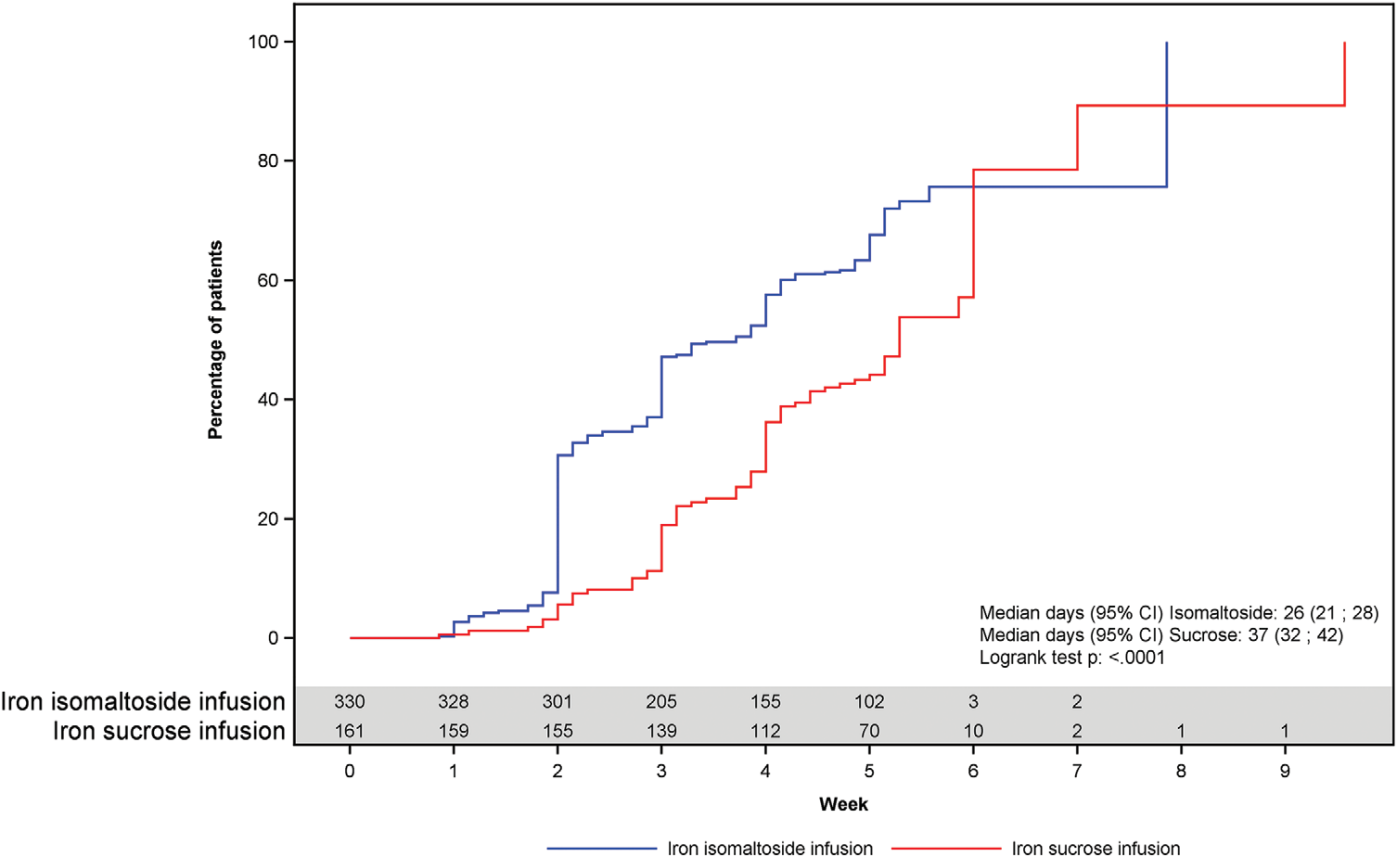
Kaplan‐Meier plot of time to increase in hemoglobin of ≥2 g/dL, full analysis set.

The median time to Hb increase ≥2 g/dL was 26 days in the iron isomaltoside group and 37 days in the iron sucrose group. A Kaplan‐Meier plot of time to increase is shown in Figure 1.

Analysis of time to Hb increase ≥2 g/dL showed a statistically significantly shorter time to Hb increase ≥2 g/dL in the iron isomaltoside group compared with the iron sucrose group; hazard ratio (HR) (95% CI) of 2.488 (1.916; 3.230) (*p* < 0.0001).

**Figure 2:**
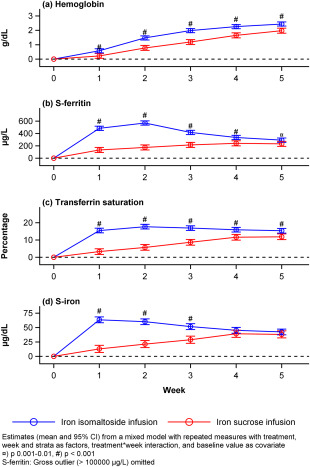
Hemoglobin, *s*‐ferritin, transferrin saturation, and *s*‐iron over time by treatment group, full analysis set.

The change from baseline in Hb was statistically significantly higher in the iron isomaltoside compared to the iron sucrose group at each time point (*p <* 0.0001) (Table 2, Figure 2), and similar results were found in the gynecology and gastroenterology subgroups (Supporting Information Figure S2).

#### 3.3.2 Change in s‐ferritin, transferrin saturation, and s‐iron

These secondary outcome analyses were conducted on the FAS population (*N* = 491).

One in the iron isomaltoside group had a *s‐*ferritin value at week 2 of >100,000 ng/mL, and an analysis was performed excluding this outlier (Supporting Information Table SIII, Figure 2). Removing this extreme value from the repeated measurement analysis showed statistically significantly higher change from baseline in *s‐*ferritin concentration in the iron isomaltoside group compared to the iron sucrose group at all time points (*p* < 0.002). An analysis including the outlier is presented in Supporting Information Table SIV. The change from baseline in TSAT and *s‐*iron was statistically significantly higher in the iron isomaltoside group compared to the iron sucrose group at each time point analyzed (*p* < 0.0001) and at weeks 1, 2, and 3 (*p* < 0.0001), respectively (Supporting Information Table SIII, Figure 2).

#### 3.3.3 Change in quality of life

The change in QoL was assessed the FAS population (*N* = 491).

In both treatment groups, the SF‐36 scores in the eight health domains as well as for the two composite scores improved from baseline to weeks 2 and 5, and there were no differences between the treatment groups (Supporting Information Figure S3).

#### 3.3.4 Safety

Safety outcomes were conducted on the safety analysis set (*N* = 501).

In the iron isomaltoside group, 75 (22.5%) reported 137 ADRs (i.e., treatment‐related adverse event), and in the iron sucrose group 29 (17.3%) reported 86 ADRs (*p* > 0.05).

More skin and subcutaneous tissue disorders were reported in the iron isomaltoside group (7.5%) than in the iron sucrose group (3.0%).

Nervous system disorders and gastrointestinal disorders were reported more frequently in the iron sucrose group than in the iron isomaltoside group. Among the nervous system disorders, dysgeusia was more common in the iron sucrose group (2.4%) than in the iron isomaltoside group (0.6%). With gastrointestinal disorders, more patients in the iron sucrose group than in the iron isomaltoside group reported nausea, vomiting, diarrhea, and dyspepsia. Fatigue was reported by 1.2% in the iron sucrose group and none in the iron isomaltoside group. Hypophosphatemia was reported as an ADR in 1.5% in the iron isomaltoside group and was not reported in the iron sucrose group.

Serious adverse reactions (SARs) (severe dyspnea and severe pruritic rash in one and moderate syncope in one) were reported by 0.6% of the patients in the iron isomaltoside group. In the iron sucrose group 0.6% also reported SARs (severe anaphylactic reaction).

One patient in the iron isomaltoside group died during the trial. The event was reported as cardiorespiratory arrest with underlying cardiac disease and was not related to trial drug.

## 4 Discussion

Outside the USA, iron isomaltoside has been approved in more than 30 countries worldwide for treatment of iron deficiency when oral iron formulations are ineffective or cannot be used or when there is a clinical need to deliver iron rapidly. The objectives of this trial were to evaluate the efficacy and safety of IV iron isomaltoside in comparison to iron sucrose in patients with IDA. The strength of the present trial was that it included a broad population with different IDA etiologies. These included a large proportion of pre‐menopausal women with menorrhagia who were otherwise healthy. Furthermore, IDA was confirmed in all patients since enrollment was based on low values of Hb, TSAT, and *s‐*ferritin (Hb <11.0 g/dL, TSAT <20%, and *s*‐ferritin <100 ng/mL). The mean cumulative dose of iron isomaltoside given was 1640.2 (SD: 357.6) mg and of iron sucrose 1127.9 (SD: 343.3) mg. The difference in cumulative doses reflects the dosing opportunities for the two IV iron products, where iron isomaltoside has an advantage over iron sucrose in requiring fewer administrations and hence a shorter treatment period to reach a higher and clinically required iron dose. The cumulative dose of iron isomaltoside was calculated by a simplified dosing formula based upon baseline Hb and weight, whereas the cumulative dose for iron sucrose was calculated by the Ganzoni formula. It needs to be noted, that the Ganzoni formula has previously been shown to underestimate iron requirements,^17^, ^27^ and in a previous reported trial in IBD patients with IDA comparing a simplified dosing regimen of ferric carboxymaltose with Ganzoni‐calculated doses of iron sucrose, the simplified dosing regimen showed a better efficacy and compliance profile.^23^

For the primary endpoint, the proportion reaching a Hb increase from baseline of ≥2 g/dL at any time between week 1 and 5, both non‐inferiority and superiority was confirmed for iron isomaltoside compared with iron sucrose. Furthermore, shorter time to Hb increase ≥2 g/dL was observed with iron isomaltoside compared to iron sucrose, which was most likely because of the fact that iron isomaltoside was given in higher doses within a shorter time period. For all biochemical efficacy parameters measured (Hb, *s‐*ferritin, TSAT, and *s‐*iron), faster and/or greater improvements were found with iron isomaltoside compared to iron sucrose. These findings are in agreement with previous trials with iron isomaltoside reporting efficacy in significantly increasing iron related parameters.^13^, ^14^, ^15^, ^16^, ^17^, ^18^, ^19^, ^20^, ^21^, ^22^

In the present trial, QoL improved in both treatment groups which was expected because of the correction of the iron deficiency. No difference between groups was found.

Treatments with iron isomaltoside and iron sucrose were well tolerated. Compared with iron isomaltoside, the iron is more loosely bound in iron sucrose.^12^ This is associated with catalytic/labile iron which has been hypothesized to cause increased oxidative stress with potential consequences on long term toxicity.^28^, ^29^ Non‐serious ADRs, especially rash and pruritus were more common with iron isomaltoside whereas dysgeusia and gastrointestinal side effects were reported more frequently with iron sucrose. SARs were reported in 0.6% in both treatment groups.

In conclusion, administration of iron isomaltoside resulted in a significantly higher and faster Hb response than did iron sucrose. Iron isomaltoside has an advantage over iron sucrose in requiring fewer administrations. Iron isomaltoside administration was efficacious and well tolerated in a broad population of IDA affected individuals.

## Conflict of interest

Lars L. Thomsen is employed by Pharmacosmos A/S, and the investigators/institutions received a fee per patient. Richard Derman has been a consultant for Pharmacosmos A/S. Michael Auerbach has received research funding from Pharmacosmos A/S and AMAG Pharmaceuticals and has consulted for Pharmacosmos A/S, AMAG Pharmaceuticals, and Luitpold Pharmaceuticals. Eloy Roman, Manuel R. Modiano, and Maureen M. Okam have no further conflicts of interest.

## Supporting information

Supporting Information
