# Supplementary material for: A randomized trial of iron isomaltoside versus iron sucrose in patients with iron deficiency anemia
Source: Am J Hematol. 2017 Feb 1;92(3):286–91. doi: 10.1002/ajh.24633 (PMC5363238; doi:10.1002/ajh.24633)

**Supplementary tables and figures**

**Table S1** Inclusion and exclusion criteria

| **Inclusion criteria:** |
| --- |
| - Men or women ≥ 18 years having iron deficiency anemia (IDA) caused by different etiologies* such as abnormal uterine bleeding, gastrointestinal diseases, cancer, bariatric procedures (gastric bypass operations), and other conditions leading to significant blood loss and with a documented history of intolerance or unresponsiveness to oral iron therapy** for at least one month*** prior to trial enrolment or where there at investigator’s judgment was a clinical need to deliver iron rapidly - Hemoglobin <11 g/dL - Transferrin saturation <20% - *S*-ferritin <100 ng/mL - Willingness to participate and signing the informed consent form   *The etiology for IDA should be documented in the medical history and verified in the source document.  **The intolerance and non-response to oral iron treatment should be documented with sign and symptoms in the medical history and verified in the source document.  ***There should be a documentation of at least one month of intolerance or unresponsiveness to oral iron therapy per investigator’s judgment within the last 2 years and they would not be candidates for oral iron again. |
| **Exclusion criteria:** |
| - Anemia predominantly caused by factors other than IDA (e.g. anemia with untreated vitamin B_12_ or folate deficiency, hemolytic anemia) - Iron overload or disturbances in utilization of iron (e.g. haemochromatosis and haemosiderosis) - Decompensated liver cirrhosis or active hepatitis (alanine aminotransferase >3 times upper limit of normal) - Active acute or chronic infections (assessed by clinical judgement supplied with white blood cells and C-Reactive Protein) - Body weight <50 kg - Rheumatoid arthritis with symptoms or signs of active inflammation - Pregnant or nursing women. In order to avoid pregnancy, women of childbearing potential had to use adequate contraception (e.g. intrauterine devices, hormonal contraceptives, or double barrier method) during the whole trial period and 7 days after the last dosing - Known hypersensitivity to parenteral iron or any excipients in the investigational drug products - Erythropoietin treatment within 8 weeks prior to the screening visit - Other intravenous iron treatment or blood transfusion within 4 weeks prior to the screening visit - Planned elective surgery during the trial - Participation in any other interventional trial within 3 months prior to screening - Any other medical condition that, in the opinion of investigator, may have caused the patient to be unsuitable for the completion of the trial or placed the patient at potential risk from being in the trial, e.g. history of multiple allergies, uncontrolled hypertension, unstable ischemic heart disease, or uncontrolled diabetes mellitus |

**Table SII** Screening and baseline laboratory variables, full analysis set

|  | **Iron isomaltoside** | | **Iron sucrose** | |
| --- | --- | --- | --- | --- |
|  | **Screening*** | **Baseline** | **Screening*** | **Baseline** |
| **Hemoglobin (g/dL)** |  |  |  |  |
| N | 330 | 330 | 161 | 161 |
| Mean (SD) | 9.5 (1.1) | 9.4 (1.2) | 9.4 (1.2) | 9.4 (1.3) |
| Median (Min; Max) | 9.7 (4.8; 10.9) | 9.5 (4.4; 12.1) | 9.7 (6.1; 10.9) | 9.7 (6.1; 12.2) |
| ***S*-ferritin (ng/mL)** |  |  |  |  |
| N | 329 | 330 | 161 | 161 |
| Mean (SD) | 13.2 (14.9) | 14.3 (32.8) | 12.3 (14.3) | 15.6 (47.2) |
| Median (Min; Max) | 8.0 (2.0; 97.0) | 7.5 (2; 543) | 7.0 (2.0; 81.0) | 8.0 (2; 581) |
| **TSAT (%)** |  |  |  |  |
| N | 330 | 330 | 161 | 161 |
| Mean (SD) | 5.2 (3.3) | 5.8 (5.0) | 5.6 (4.0) | 6.4 (5.9) |
| Median (Min; Max) | 4.0 (1.0; 19.0) | 4.0 (1.0; 43.0) | 4.0 (1.0; 19.0) | 4.0 (1.0; 40.0) |

*The patients were enrolled in the trial based on screening values for Hb, *s-*ferritin, and TSAT.

**Table SIII** Change from baseline in hemoglobin, *s*-ferritin, transferrin saturation, and *s*-iron, full analysis set

| **Laboratory variables, time point (number of patients)** | **Iron isomaltoside (group A), least-square mean estimate^a^** | **Iron sucrose (group B), least-square mean estimate^a^** | **Difference estimates (95% CI)** | ***P* value^a^** |
| --- | --- | --- | --- | --- |
| **Hemoglobin (g/dL)** | | | | |
| Week 1 (group A: 319, group B: 153) | 0.58 | 0.23 | 0.35 (0.19; 0.52) | <0.0001 |
| Week 2 (group A: 318, group B: 157) | 1.48 | 0.78 | 0.70 (0.53; 0.86) | <0.0001 |
| Week 3 (group A: 311, group B: 150) | 1.98 | 1.19 | 0.79 (0.63; 0.96) | <0.0001 |
| Week 4 (group A: 317, group B: 148) | 2.26 | 1.65 | 0.60 (0.44; 0.77) | <0.0001 |
| Week 5 (group A: 322, group B: 155) | 2.44 | 1.98 | 0.46 (0.30; 0.62) | <0.0001 |
| ***S-*ferritin (ng/mL)^b^** | | | | |
| Week 1 (group A: 326, group B: 159) | 484 | 135 | 349 (312; 386) | <0.0001 |
| Week 2 (group A: 321, group B: 159) | 571 | 174 | 397 (360; 434) | <0.0001 |
| Week 3 (group A: 319, group B: 153) | 418 | 215 | 203 (166; 240) | <0.0001 |
| Week 4 (group A: 321, group B: 150) | 337 | 241 | 95.5 (58; 133) | <0.0001 |
| Week 5 (group A: 323, group B: 155) | 292 | 234 | 59 (22; 96) | 0.0019 |
| **Transferrin saturation (%)** | | | | |
| Week 1 (group A: 318, group B: 153) | 15.5 | 3.3 | 12.2 (10.6; 13.8) | <0.0001 |
| Week 2 (group A: 322, group B: 152) | 17.7 | 5.7 | 12.0 (10.4; 13.6) | <0.0001 |
| Week 3 (group A: 316, group B: 148) | 16.9 | 8.7 | 8.2 (6.6; 9.8) | <0.0001 |
| Week 4 (group A: 320, group B: 147) | 16.0 | 11.6 | 4.4 (2.7; 6.0) | <0.0001 |
| Week 5 (group A: 323, group B: 155) | 15.4 | 11.9 | 3.5 (1.9; 5.1) | <0.0001 |
| ***S-*iron (µg/dL)** | | | | |
| Week 1 (group A: 323, group B: 155) | 63.5 | 13.1 | 50.4 (44.3; 56.6) | <0.0001 |
| Week 2 (group A: 322, group B: 154) | 60.2 | 21.4 | 38.8 (32.6; 44.9) | <0.0001 |
| Week 3 (group A: 316, group B: 151) | 51.7 | 28.9 | 22.8 (16.6; 29.0) | <0.0001 |
| Week 4 (group A: 320, group B: 150) | 45.3 | 39.2 | 6.1 (-0.1; 12.2) | 0.0556 |
| Week 5 (group A: 321, group B: 155) | 42.4 | 38.2 | 4.2 (-1.9; 10.4) | 0.1777 |

^a^The estimates are from a mixed model for repeated measures (MMRM) with treatment, visit, treatment-by-visit, and stratum as factors and baseline value as covariate.

^b^For one patient in the iron isomaltoside group a value at week 2 of >100000 ng/mL was removed.

Conversion factor for *s-*iron: µmol/L / 0.179 = µg/dL

**Table SIV** Change from baseline in *s-*ferritin (full analysis set with outlier included)

| **Laboratory variables, time point (number of patients)** | **Iron isomaltoside (group A), least-square mean estimate^a^** | **Iron sulfate (group B), least-square mean estimate^a^** | **Difference estimates (95% CI)** | ***P* value^a^** |
| --- | --- | --- | --- | --- |
| ***S-*ferritin (ng/mL)** | | | | |
| Week 1 (group A: 326, group B: 159) | 466 | 117 | 349 (-39; 737) | 0.0782 |
| Week 2 (group A: 322, group B: 159) | 860 | 157 | 703 (314; 1092) | 0.0004 |
| Week 3 (group A: 319, group B: 153) | 402 | 200 | 202 (-192; 597) | 0.3149 |
| Week 4 (group A: 321, group B: 150) | 319 | 224 | 95 (-302; 492) | 0.6398 |
| Week 5 (group A: 323, group B: 155) | 276 | 218 | 59 (-334; 451) | 0.7700 |

^a^The estimates are from a mixed model for repeated measures (MMRM) with treatment, visit, treatment-by-visit, and stratum as factors and baseline value as covariate.

**Figure S1** Patient disposition


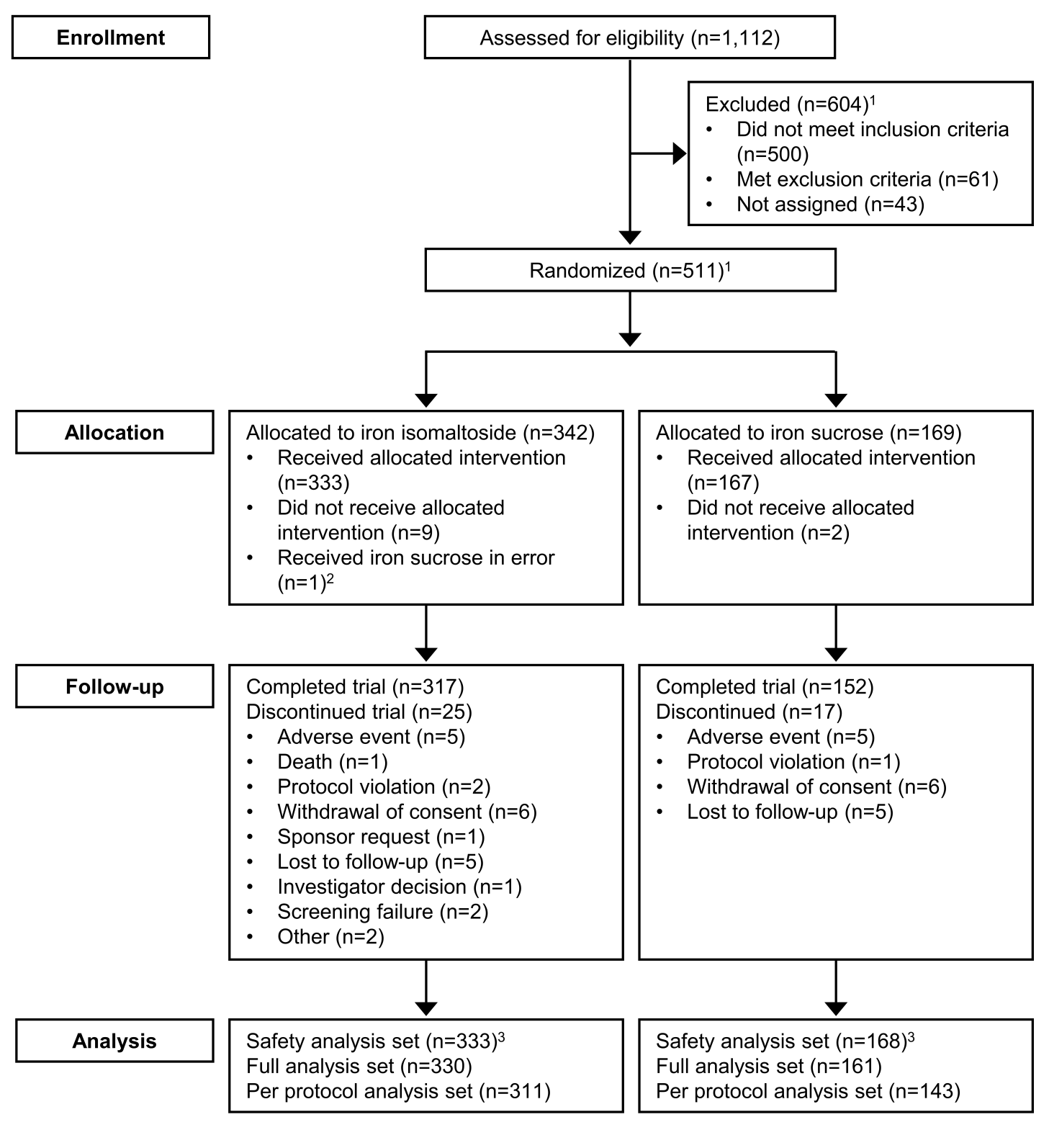


^1^Three patients who failed screening were randomised in error; two patients in the iron isomaltoside group were later withdrawn, and one patient completed the trial.

^2^One patient was randomised to iron isomaltoside but treated with iron sucrose.

^3^The safety analysis set was counted based on actual treatment.

**Figure S2** Change from baseline in hemoglobin in the gynecology and gastroenterology subgroups, full analysis set

**
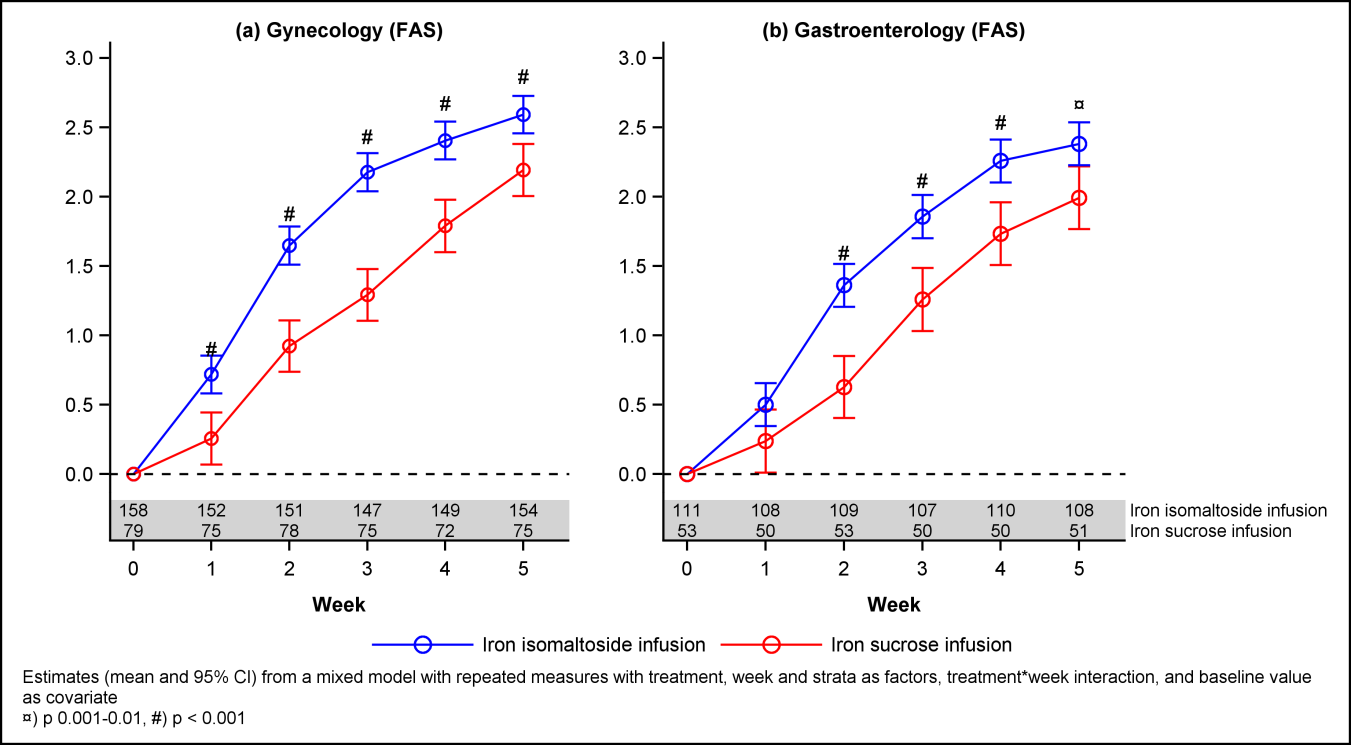
**

**Figure S3** QoL measured by the SF-36 score, full analysis set


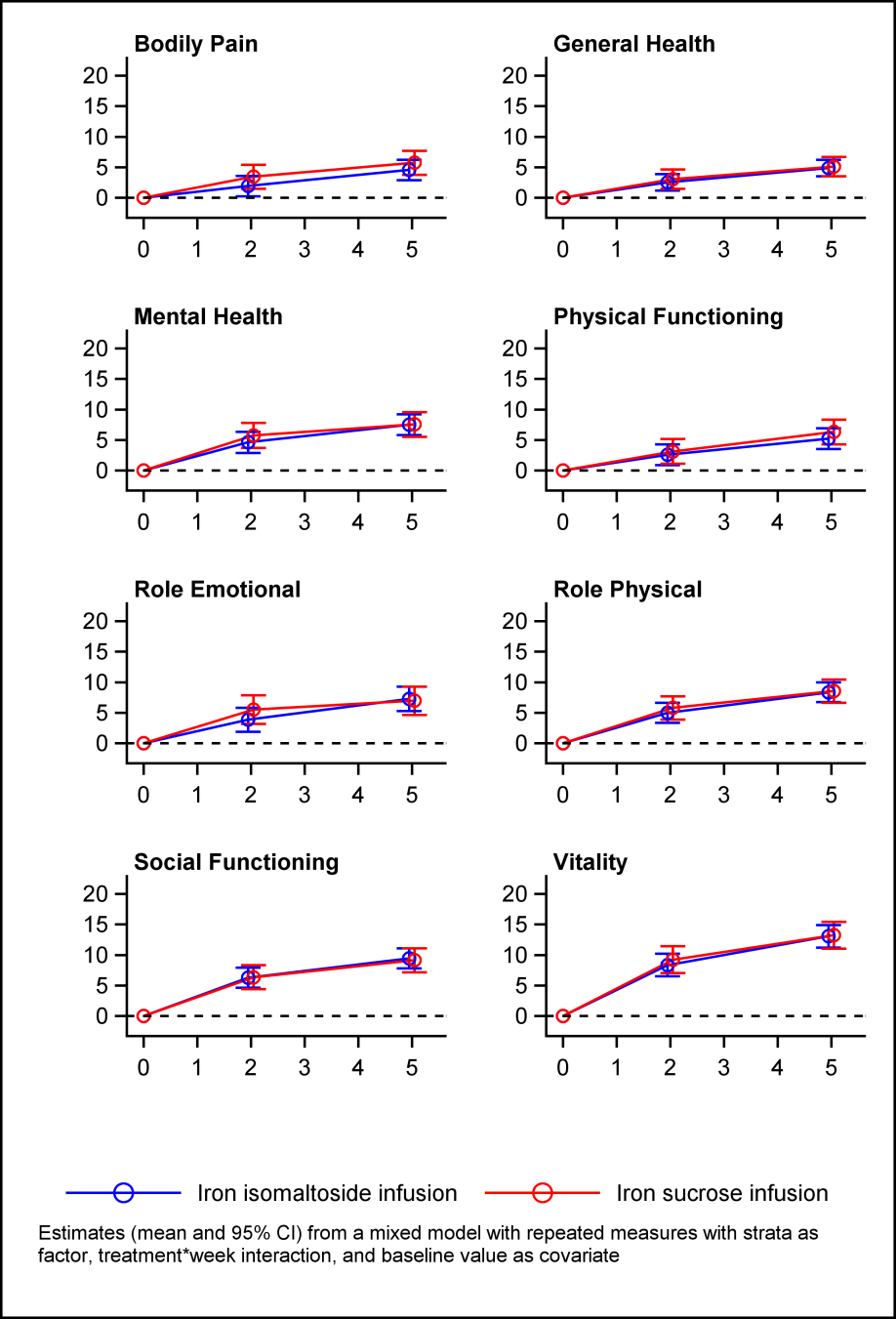

Supplement: Supplementary file 1 — Supporting Information [file AJH-92-286-s001.docx]
